# Efficacy and safety of sotagliflozin adjuvant therapy for type 1 diabetes mellitus: A systematic review and meta-analysis

**DOI:** 10.1097/MD.0000000000020875

**Published:** 2020-08-14

**Authors:** Mao-Bing Chen, Rui-Jun Xu, Qi-Han Zheng, Xu-Wen Zheng, Hua Wang

**Affiliations:** a Department of Emergency; b Department of Endocrinology; c Department of ICU, Wujin People Hospital Affiliated with Jiangsu University, and the Wujin Clinical College of Xuzhou Medical University, Changzhou, Jiangsu, P. R. China.

**Keywords:** meta-analysis, randomized controlled trial, sodium-dependent glucose transporter-1 inhibitors, sodium-dependent glucose transporter-2 inhibitors, sotagliflozin, type 1 diabetes mellitus

## Abstract

**Background:**

To systematically evaluate the efficacy and safety of sotagliflozin (SOTA) adjuvant therapy for type 1 diabetes mellitus (T1DM).

**Methods:**

Through April 2019, the Web of Science, PubMed, Cochrane Library, Embase, and China National Knowledge Infrastructure databases were electronically searched to identify randomized controlled trials exploring SOTA adjuvant therapy for T1DM. Strict screening and quality evaluations of the obtained literature were performed independently by 2 researchers. Outcome indexes were extracted, and a meta-analysis of the data was performed using Revman 5.3 software.

**Results:**

A total of 7 randomized controlled trials were included. The meta-analysis results showed that compared with the patients in the placebo group, the patients in the SOTA group had a lower hemoglobin A1c (mean difference [MD] = −0.28, 95% confidence interval [CI] [−0.34, −0.22], *P* < .01), lower total daily insulin use (MD = −8.89, 95% CI [−11.64, −6.13], *P* < .01), faster weight loss (MD = −3.03, 95% CI [−3.79, −2.26], *P* < .01), better fasting blood glucose and 2-hour postprandial blood glucose control (MD = −0.75, 95% CI [−1.04, −0.45], *P* < .01; MD = −2.42, 95% CI [−3.17, −1.67], *P* < .01), and a higher rate of well-controlled glucose levels (relative risk = 1.75, 95% CI [1.55, 1.99], *P* < .01), while no significant difference in the incidence of severe hypoglycemic events was found between the SOTA and placebo groups (risk difference [RD] = −0.01, 95% CI [−0.02, 0.00], *P* = .13). The incidence of diabetic ketoacidosis was higher in the SOTA group than in the placebo group (RD = 0.03, 95% CI [0.02, 0.04], *P* < .01). The incidence of genital mycotic infection was higher in the SOTA group than in the placebo group (RD = 0.06, 95% CI [0.05, 0.08], *P* < .01). No significant difference in the incidence of urinary tract infections was detected between the SOTA group and the placebo group (RD = 0.00, 95% CI [−0.01, 0.01], *P* = 0.97).

**Conclusions:**

SOTA is a potential drug for the treatment of T1DM and is effective for controlling blood sugar. The main adverse reactions to SOTA are genital mycotic infections and diabetic ketoacidosis. We must further assess the severity of diabetic ketoacidosis caused by SOTA.

## 1 Introduction

Type 1 diabetes mellitus (T1DM), or insulin-dependent diabetes, is most common in children and adolescents, affecting millions of people worldwide.^[1]^ Similar to hypertension, diabetes is a major cause of mortality and may have atypical presentations such as sexual dysfunction and coma, which can occur due to diabetic ketoacidosis (DKA).^[2,3]^ In recent years, diabetes has been found to seriously damage human health. Although China still has one of the lowest rates of T1DM in the world, in the past 20 years, the incidence of T1DM among children under the age of 15 years has nearly quadrupled, and the number of newly diagnosed adults with T1DM has increased significantly.^[4]^ Owing to insufficient insulin production, patients must submit to multiple daily injections of insulin or continuous subcutaneous insulin injection; otherwise, blood glucose cannot be well controlled.^[5]^ The incidence of T1DM is lower than that of type 2 diabetes mellitus (T2DM), but T1DM is more dangerous. Individuals with T1DM are prone to serious complications that can sometimes be life-threatening, such as severe hypoglycemia, hypertonic coma, and DKA.^[6]^

Few noninsulin-associated therapies are available for the treatment of T1DM. Sodium-dependent glucose transporter-2 (SGLT-2) inhibitors have been a popular topic in research on diabetes drugs in recent years.^[7]^ SGLT-2 regulates blood glucose through the excretion function of the kidneys in addition to the metabolic pathway of glucose in the body by means of increasing the excretion of glucose by the kidneys.^[8]^ SGLT-2 inhibitors are approved in many countries to treat diabetes.

Sotagliflozin (SOTA) is a novel SGLT-1/SGLT-2 dual inhibitor. Relying on its unique hypoglycemic mechanism, it reduces the absorption of glucose in the gastrointestinal tract by inhibiting SGLT-1 and increases the excretion of glucose by the kidneys by inhibiting SGLT-2. Studies have found that SOTA can not only treat T2DM but can also treat T1DM.^[9]^ Thus far, SOTA has passed several phase 4 clinical trials (inTandem1, inTandem2, inTandem3, and inTandem4).

The purpose of this meta-analysis is to analyze the therapeutic effect and safety of SOTA on T1DM, thereby providing evidence for the treatment of T1DM by SOTA.

## 2 Methods

### 2.1 Design and registration

A meta-analysis will be conducted to evaluate the efficacy and safety of SOTA adjuvant therapy for T1DM. This protocol has been registered on the international prospective register of systematic reviews (PROSPERO), registration number: CRD42019133099 (https://www.crd.york.ac.uk/PROSPERO). No ethical approval is required since this study used data that were already in the public domain.

### 2.2 Study selection

#### 2.2.1 Study type

All the trials in this meta-analysis were randomized controlled trials (RCTs).

#### 2.2.2 Study object

Type 1 diabetic patients who rely on insulin to control their glucose using multiple daily injections or continuous subcutaneous insulin injection to inject insulin, excluding individuals with other serious underlying diseases.

#### 2.2.3 Intervening measure

Patients received treatment for a period of time to stabilize their blood glucose and glycosylated hemoglobin (HbAlc) before the experiment. In the case of normal insulin therapy, SOTA tablets or placebo should be taken once a day.

#### 2.2.4 Outcome indicators

The following outcomes were assessed and compared with the effects of the placebo:

(1) HbAlc,
(2) the total daily insulin dose (TDD),
(3) weight,
(4) fasting blood glucose,
(5) (5) 2-hour postprandial blood glucose,
(6) well-controlled diabetes (HbAlc <7% after the end of the study without severe hypoglycemia or DKA),
(7) severe hypoglycemia,
(8) DKA,
(9) genital mycotic infections, and
(10) urinary tract infections.

#### 2.2.5 Exclusion criteria

Literature whose data cannot be extracted or utilized; literature on animal experiments; literature reviews, and so on.

### 2.3 Data sources and searches

**Figure 1:**
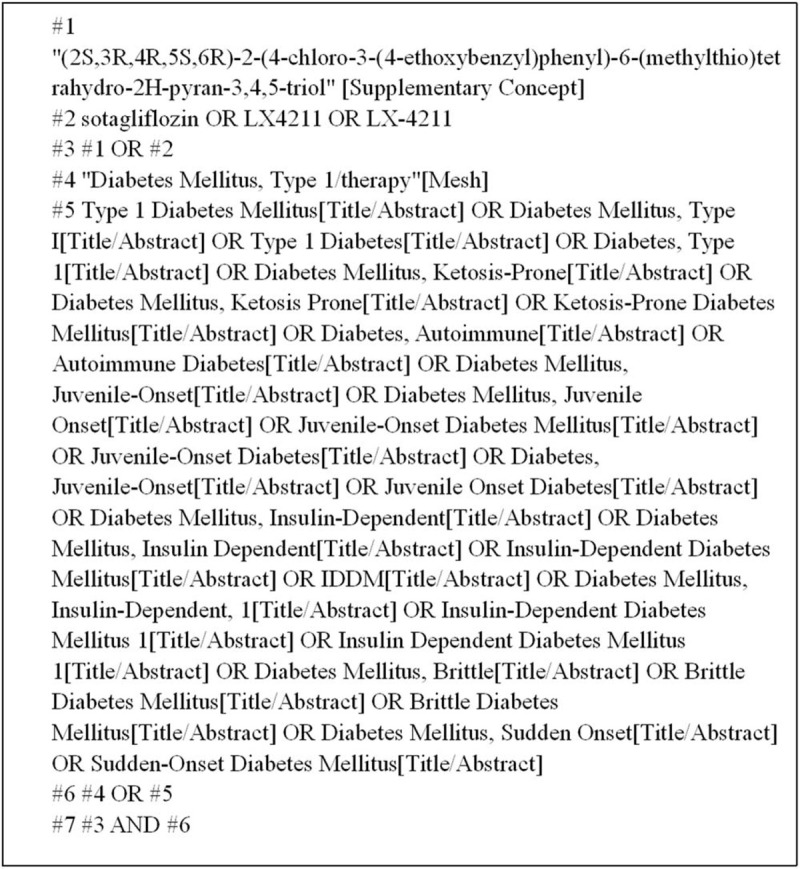
PubMed database retrieval strategy.

We searched English and Chinese language publications through April 2019 using the following databases: Web of Science, PubMed, the Cochrane Library, Embase, and the China National Knowledge Infrastructure. The search terms included “sotagliflozin,” “Type 1 Diabetes Mellitus,” “T1DM,” “LX4211” and so on. Here, we use the PubMed database as an example (Fig. 1).

### 2.4 Study screening, data extraction, and risk assessment of bias

Data were collected independently by 2 researchers. The unqualified studies were eliminated, and the qualified ones were screened out after reading the title, abstract and full text. Then, the research data were extracted and checked, and disagreements were discussed or a decision was made by the author. The extracted data included the following:

(1) basic information of the study, including title, author and year of publication;
(2) characteristics of the included study, consisting of study duration, sample size of test group and control group, and intervention measures;
(3) outcome indicators and data included; and
(4) collection of risk assessment elements of bias.

The risk of bias in the included studies was assessed by using the RCT bias risk assessment tool recommended in the Cochrane Handbook for Systematic Reviews of Interventions (5.1.0).

### 2.5 Statistical analysis

Revman 5.3 software was used for the meta-analysis. The dichotomous variables were relative risk (RR) or risk difference (RD) as effect indicators, the continuous variables are expressed as mean difference (MD) as effect indicators, and the estimated value and 95% confidence interval (CI) were included as effect analysis statistics. A heterogeneity test was conducted with the results of each study. The fixed effect model was used for analysis if there was no statistical heterogeneity between the results (*I*^2^ ≤ 50%). The sources of heterogeneity needed to be analyzed if there was statistical heterogeneity between the results (*I*^2^ > 50%). After excluding the influence of obvious clinical heterogeneity, the random effect model was used for analysis. The significance level was set α = 0.05.

## 3 Results

### 3.1 Retrieved results

**Figure 2:**
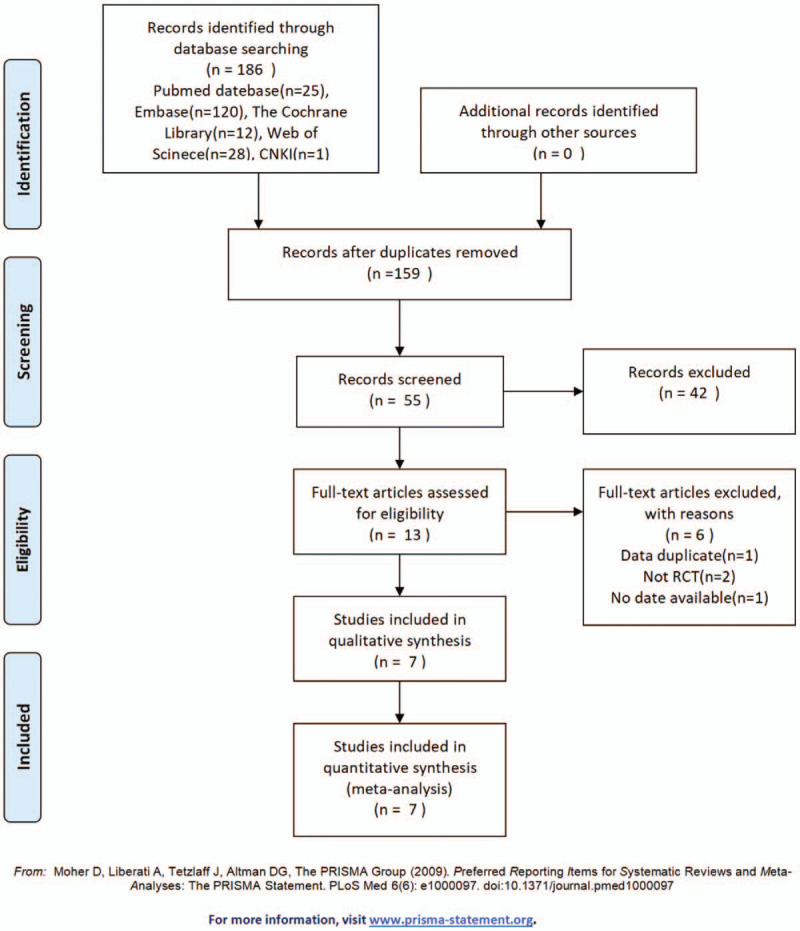
Flow diagram of evidence acquisition during the study.

A total of 186 studies were initially selected, and 7^[10–16]^ studies were finally included after screening; all of the included studies were written in English. The literature screening process and results are shown in Figure 2.

### 3.2 Basic information of studies

The basic characteristics of the included studies are shown in Table 1, and the bias risk evaluation results are shown in Table 2.

**Table 1:**
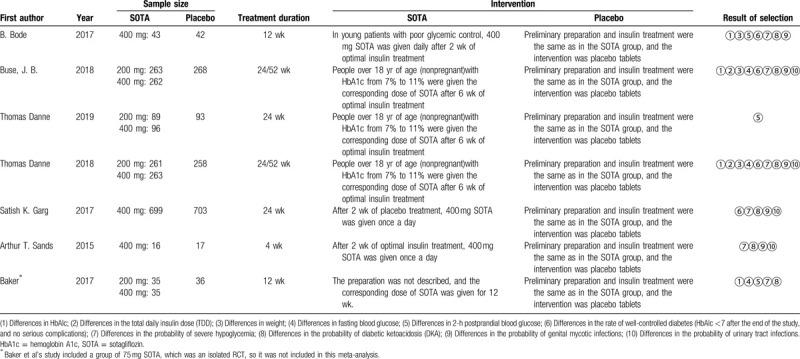
Basic information of the study.

**Table 2:**
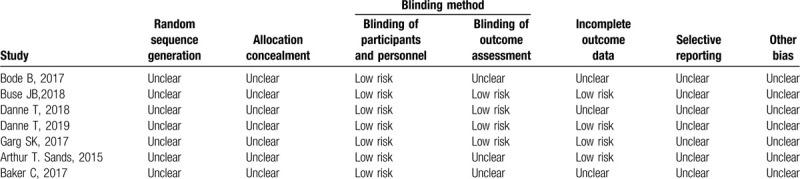
Bias risk assessment included in the study.

### 3.3 Meta-analysis results

Seven studies were included in this study, and a total of 3479 individuals were included, including 648 patients who received 200 mg of SOTA orally, 1414 patients who received 400 mg of SOTA orally, and 1417 patients who received placebo orally. This study collected data on the differences between the results of the SOTA group and the placebo group and extracted or calculated the MD and standard error (SE) from the literature. In this study, the oral dose of SOTA was used as the grouping standard for the subgroup analysis, and subgroups were established based on the oral doses of 200 mg SOTA and 400 mg SOTA. The outcome indexes of multiple timepoints appear in the literature. The indexes were analyzed at the end of the test since the data of intermediate timepoints could not be extracted completely.

#### 3.3.1 HbAlc

**Figure 3:**
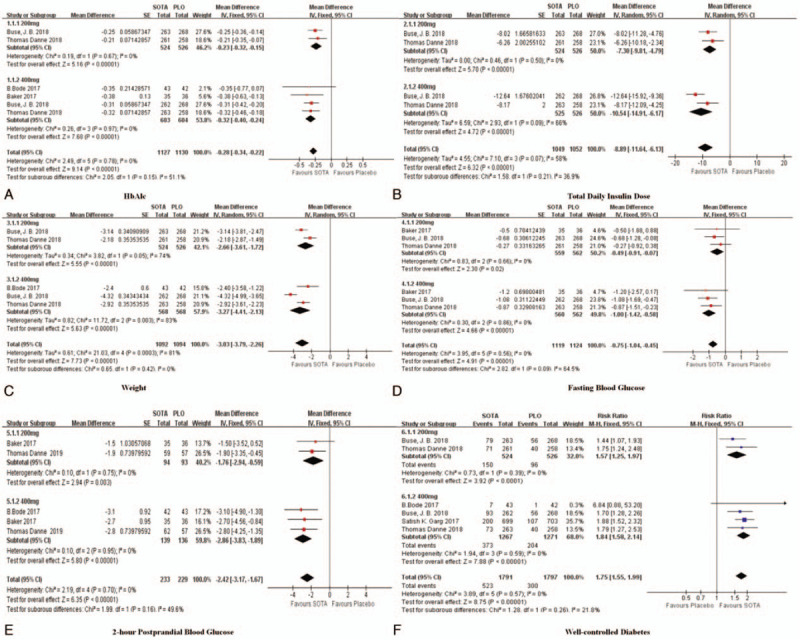
Forest plot comparing the effects of SOTA versus the placebo on efficacy. SOTA = sotagliflozin.

Five studies reported differences in HbAlc between the SOTA group and the placebo group. There were 1127 patients in the SOTA group and 1130 patients in the placebo group. A fixed effect model was adopted, and the HbAlc in the SOTA group was lower than that in the placebo group (oral administration of 200 mg of SOTA subgroup: *I*^2^ = 0% [MD = 0.23, 95% CI (0.32, 0.15), *P* < .01], oral administration of 400 mg of SOTA subgroup: *I*^2^ = 0% [MD = 0.32, 95% CI (0.40, 0.24), *P* < .01], all studies: *I*^2^ = 0% [MD = 0.28, 95% CI (0.34, 0.22), *P* < .01]) (Fig. 3).

#### 3.3.2 TDD

Two studies reported differences in TDD between the SOTA group and the placebo group. There were 1049 patients in the SOTA group and 1052 patients in the placebo group. In the 400 mg SOTA subgroup (*I*^2^ > 50%) the effect may be related to the duration of oral SOTA administration. A random effect model was adopted, and the TDD in the SOTA group was lower than that in the placebo group (oral administration of 200 mg of SOTA subgroup: *I*^2^ = 0% [MD = −7.30, 95% CI (−9.81, −4.79), *P* < .01], oral administration of 400 mg of SOTA subgroup: *I*^2^ = 66% [MD = −10.54, 95% CI (−14.91, −6.17), *P* < .01], all studies: *I*^2^ = 58% [MD = −8.89, 95% CI (−11.64, −6.13), *P* < .01]) (Fig. 3).

#### 3.3.3 Weight

Two studies reported differences in weight between the SOTA group and the placebo group. There were 1092 patients in the SOTA group and 1094 patients in the placebo group. In the 200 mg SOTA and 400 mg SOTA subgroups (*I*^2^ > 50%), the effects may be related to the duration of oral SOTA. The longer the patients took SOTA, the more weight they may have lost. A random effect model was adopted, and the weight loss was greater in the SOTA group than in the placebo group (oral administration of 200 mg of SOTA subgroup: *I*^2^ = 74% [MD = −2.66, 95% CI (−3.61, −1.72), *P* < .01], oral administration of 400 mg of SOTA subgroup: *I*^2^ = 83% [MD = −3.27, 95% CI (−4.41, −2.31), *P* < .01], all studies: *I*^2^ = 81% [MD = −3.03, 95% CI (−3.79, 2.26), *P* < .01]) (Fig. 3).

#### 3.3.4 Fasting blood glucose

Three studies reported differences in fasting blood glucose between the SOTA group and the placebo group. There were 1119 patients in the SOTA group and 1124 patients in the placebo group. A fixed effect model was adopted, and fasting blood glucose was shown to be better controlled in the SOTA group than in the placebo group (oral administration of 200 mg of SOTA subgroup: *I*^2^ = 0% [MD = −0.49, 95% CI (−0.91, −0.07), *P* = .02], oral administration of 400 mg of SOTA subgroup: *I*^2^ = 0% [MD = −1, 95% CI (−1.42, 0.58), *P* < .01], all studies: *I*^2^ = 0% [MD = −0.75, 95% CI (−1.04, −0.45), *P* < .01]) (Fig. 3).

#### 3.3.5 Two-hour postprandial blood glucose

Three studies reported differences in 2-hour postprandial blood glucose between the SOTA group and the placebo group. There were 233 patients in the SOTA group and 229 patients in the placebo group. A fixed effect model was adopted, and 2-hour postprandial blood glucose was better controlled in the SOTA group than in the placebo group (oral administration of 200 mg of SOTA subgroup: *I*^2^ = 0% [MD = −1.76, 95% CI (−2.94, −0.59), *P* = .003], oral administration of 400 mg of SOTA subgroup: *I*^2^ = 0% [MD = −2.86, 95% CI (−3.83, −1.89), *P* < .01], all studies: *I*^2^ = 0% [MD = −2.42, 95% CI (−3.17, −1.67), *P* < .01]) (Fig. 3).

#### 3.3.6 Well-controlled diabetes

Four studies reported differences in the rate of well-controlled diabetes between the SOTA group and the placebo group. There were 1791 patients in the SOTA group and 1,797 patients in the placebo group. A fixed effect model was adopted, and in the SOTA group, more patients had well controlled diabetes than in the placebo group (oral administration of 200 mg of SOTA subgroup: *I*^2^ = 0% [RR = 1.57, 95% CI (1.25, 1.97), *P* < .01], oral administration of 400 mg of SOTA subgroup: *I*^2^ = 0% [RR = 1.84, 95% CI (1.58, 2.14), *P* < .01], all studies: *I*^2^ = 0% [RR = 1.75, 95% CI (1.55, 1.99), *P* < .01]) (Fig. 3).

#### 3.3.7 Severe hypoglycemia

**Figure 4:**
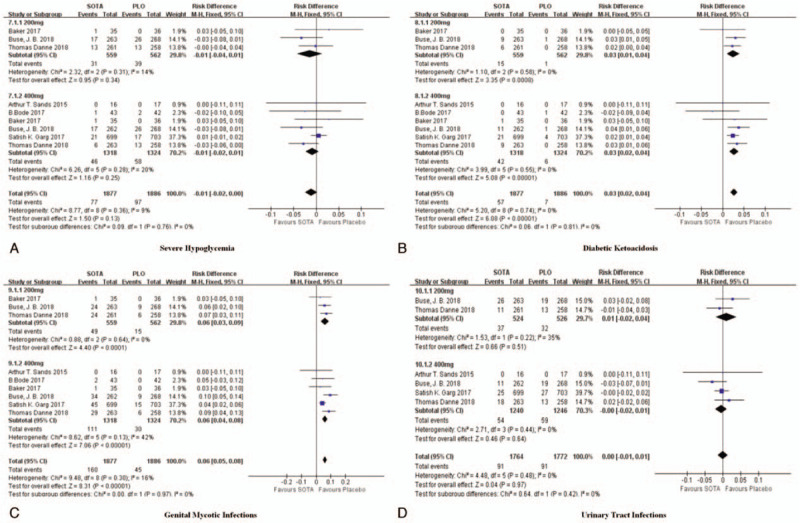
Forest plot comparing effects of SOTA versus the placebo on safety. SOTA = sotagliflozin.

Six studies reported differences in the probability of severe hypoglycemia between the SOTA group and the placebo group. There were 1877 patients in the SOTA group and 1886 patients in the placebo group. A fixed effect model was adopted, and there was no statistically significant difference in the incidence of severe hypoglycemic events between the SOTA group and the placebo group (oral administration of 200 mg of SOTA subgroup: *I*^2^ = 14% [RD = −0.01, 95% CI (−0.04, 0.01), *P* = .34], oral administration of 400 mg of SOTA subgroup: *I*^2^ = 20% [RD = −0.01, 95% CI (−0.02, 0.01), *P* = .25], all studies: *I*^2^ = 9% [RD = −0.01, 95% CI (−0.02, 0.00), *P* = .13]) (Fig. 4).

#### 3.3.8 DKA

Six studies reported differences in the probability of DKA between the SOTA group and the placebo group. There were 1877 patients in the SOTA group and 1886 patients in the placebo group. A fixed effect model was adopted, and the SOTA group had a higher incidence of DKA events than the placebo group (oral administration of 200 mg of SOTA subgroup: *I*^2^ = 0% [RD = 0.03, 95% CI (0.01, 0.04), *P* < .01], oral administration of 400 mg of SOTA subgroup: *I*^2^ = 0% [RD = 0.03, 95% CI (0.02, 0.04), *P* < .01], all studies: *I*^2^ = 0% [RD = 0.03, 95% CI (0.02, 0.04), *P* < .01]) (Fig. 4).

#### 3.3.9 Genital mycotic infections

Six studies reported differences in the probability of genital mycotic infections between the SOTA group and the placebo group. There were 1877 patients in the SOTA group and 1886 patients in the placebo group. A fixed effect model was adopted, and the SOTA group had a higher incidence of genital mycotic infection events than the placebo group (oral administration of 200 mg of SOTA subgroup: *I*^2^ = 0% [RD = 0.06, 95% CI (0.03, 0.09), *P* < .01], oral administration of 400 mg of SOTA subgroup: *I*^2^ = 42% [RD = 0.06, 95% CI (0.04, 0.08), *P* < .01], all studies: *I*^2^ = 16% [RD = 0.06, 95% CI (0.05, 0.08), *P* < .01]) (Fig. 4).

#### 3.3.10 Urinary tract infections

Four studies reported differences in the probability of urinary tract infections between the SOTA group and the placebo group. There were 1764 patients in the SOTA group and 1772 patients in the placebo group. A fixed effect model was adopted, and there was no statistically significant difference in the incidence of urinary tract infection events between the SOTA group and the placebo group (oral administration of 200 mg of SOTA subgroup: *I*^2^ = 35% [RD = 0.01, 95% CI (−0.02, 0.04), *P* = .51], oral administration of 400 mg of SOTA subgroup: *I*^2^ = 42% [RD = −0.00, 95% CI (−0.02, 0.01), *P* = .64], all studies: *I*^2^ = 0% [RD = 0.00, 95% CI (−0.01, 0.01), *P* = .97] (Fig. 4).

## 4 Discussion

**Figure 5:**
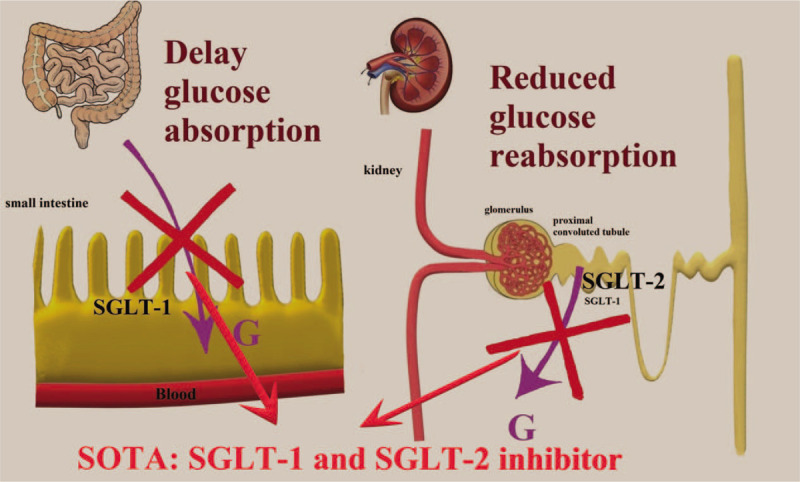
The mechanism of SOTA. SOTA = sotagliflozin.

Treating T1DM with oral drugs must be one of the directions for future drug development because of convenience and safety. SGLT-2 inhibitors have been approved for T2DM in many countries, and their efficacy and safety have been widely recognized.^[7]^ However, the efficacy and safety of SOTA adjuvant therapy for T1DM remain controversial. In Europe, SOTA has been approved for the treatment of T1DM, but the FDA rejected its use for T1DM therapy. SOTA is a new generation SGLT inhibitor that can act on both SGLT-1 and SGLT-2. SGLT-1 is mainly expressed in the small intestine and kidneys and is responsible for transporting glucose and galactose in the small intestine and reabsorbing glucose in the proximal convoluted tubules. SGLT-2 is specifically located in the proximal convoluted tubules of the kidney and is responsible for the renal reabsorption of glucose in the urine and is responsible for approximately 90% of glucose reabsorption^[17]^ (Fig. 5).

In this study, long-term oral SOTA was shown to reduce the HbAlc, fasting blood glucose, and 2-hour postprandial blood glucose of patients with T1DM, and its hypoglycemic effect was significant. T1DM patients’ islet B cells have lost the function of insulin production, and blood glucose regulation remains dependent on exogenous insulin. Before SOTA was discovered, oral hypoglycemic drugs always led to increased weight, thus increasing the risk of cardiovascular disease.^[18]^ A study found that after single administration of 300 mg of SOTA, 44 g of glucose was excreted in the urine in 24 hours.^[19]^ Sugar excreted through the urine can reduce a patient's required insulin dose and; therefore, reduce the patient's dependence on insulin.

Second, we found that compared with T1DM patients who were taking the same dose of SOTA at week 52, more patients reached the standard HbAlc level at week 24. The HbAlc measurements showed a similar trend. Although this phenomenon has been observed in several RCTs, supporting medical evidence remains insufficient. The hypoglycemic effect of SOTA seems to become weaker over time. Similar phenomena have been observed with other hypoglycemic drugs. The effect of acquired drug resistance on T1DM in the long term requires long-term follow-up investigations.

In this study, it was concluded that oral administration of SOTA did not increase the probability of severe hypoglycemia or urinary tract infections in T1DM patients. SOTA itself does not directly participate in glucose metabolism or blood glucose regulation mechanisms in the human body and has little impact on the variation in blood glucose in the human body.^[20]^ SOTA increases the concentration of glucose in the urine, theoretically increasing the likelihood of urinary tract infections. In previous studies on SGLT-2 inhibitors, relevant literature noted that under the guidance of doctors, the probability of urinary tract infections can be reduced by strengthening personal hygiene practices,^[21]^ which may explain why we did not observe more urinary tract infections with oral SOTA use in patients with T1DM. The incidence of genital mycotic infection increased after oral administration of SOTA, and the increase was statistically significant. Differences between bacterial and mycotic infections in the urinary system and the damage caused by mycotic infections require further study.

As a dual SGLT-1/SGLT-2 inhibitor, SOTA also results in the increased DKA risk associated with SGLT-2 inhibitors. SGLT-2 inhibitors can increase glucagon and the oxidation of fatty acids, reduce the clearance of ketone bodies by the kidneys, and increase the probability of DKA.^[22]^ Severe DKA can cause coma, circulatory failure, and even death.^[23]^ According to the results of the current clinical trial, the RR of DKA was 5.82, and the RD was 0.03, suggesting that the probability of DKA with oral SOTA use is approximately 5.82-times higher than that with non-oral SOTA use, and that the probability of DKA is increased by approximately 3%. Oral SOTA significantly increases the probability of DKA in patients with T1DM. Currently, T1DM can be treated with insulin, and SOTA is not irreplaceable. Serious adverse events induced by SOTA must be considered, although different views exist. In regard to the finding that SGLT-2 inhibitors result in a higher probability of DKA, which is supported by the American Association of Clinical Endocrinologists and the American College of Endocrinology, the connection between the SGLT-2 inhibitor and DKA may not have been closely reflected by the results of the study as most occurrences of DKA may be caused only by ketosis matter (ketones) accumulation; the SGLT-2 inhibitor mechanism is the result of early impacts on fat metabolism.^[24]^ Since each RCT provided only the number of DKA events and not the severity of each DKA event, future studies are needed to provide more data.

Because SOTA is a new drug, research data are limited. Sample sizes, intervention durations, SOTA doses, and inclusion criteria differed in each study, which may lead to bias in this study. This meta-analysis aimed to identify a proper balance between data integrity and data heterogeneity. We believe that the oral SOTA dose must be compared in subgroup analyses. Of course, as more RCTs are published, we hope that future studies will use uniform inclusion criteria, similar sample sizes, and the same dosages and durations for interventions. By combining homogeneous studies, the evidence in this study will be more convincing.

In the included literature, we also found that SOTA could improve patients’ systolic blood pressure and was generally well tolerated. However, the risk of SOTA use by women who are preparing for pregnancy or during pregnancy has not been studied. The study was conducted on adults, and more clinical studies are needed to verify SOTA's effectiveness and safety in children.

Limitations of this meta-analysis:

(1) In the extraction of continuous variables, different outcome indicators were provided, including SE, *P*-value, 95% CI, and so on, and the data for SE were uniformly calculated for the meta-analysis, but SE could not be directly calculated from the original data.
(2) In this analysis, the difference in the dose of SOTA was used as the basis for subgroup grouping. However, owing to the limited number of studies and data that could not be extracted, the difference in the duration of medication use was not considered. As a consequence, outcome indicators such as body weight and the rate of well-controlled diabetes may be greatly affected by the duration of medication use. If possible, subgroup analysis can be performed with different methods for 1 or 2 outcome indicators in the future to determine whether some clinically significant results can be obtained.
(3) Data on adverse reaction events with small probability need a larger sample size data to be more reliable.
(4) The sample size in each RCTs varies substantially, and the small sample sizes in the RCTs may introduce more bias.^[25]^ A better strategy to overcome this problem is to perform a meta-epidemiological study to investigate whether the sample size will influence the result.

As a new adjuvant treatment for T1DM, SOTA is in phase 4 clinical trials for T1DM. SOTA is effective for controlling blood sugar. The prominent adverse reactions include genital mycotic infections and DKA. We still need to study DKA caused by SOTA to assess the damage induced by this adverse event. We think that SOTA is still a potential treatment for T1DM.

## Acknowledgments

At the point of finishing this paper, I would like to express my sincere gratitude to all those who have lent me assistance in the course of writing this paper. I would like to express my gratitude to my workmates who offered me references and information in a timely fashion. I would like to thank the leaders, teachers, and staff, especially at my alma mater, Nanjing Medical University. Without their help, it would have been much harder for me to finish my study and this paper.

## Author contributions

**Conceptualization:** Mao-Bing Chen, Rui-Jun Xu.

**Data curation:** Mao-Bing Chen, Qi-Han Zheng, Xu-wen Zheng, Hua Wang.

**Formal analysis:** Mao-Bing Chen.

**Methodology:** Mao-Bing Chen, Hua Wang.

**Software:** Mao-Bing Chen, Xu-wen Zheng.

**Supervision:** Mao-Bing Chen, Qi-Han Zheng.

**Writing – original draft:** Mao-Bing Chen, Rui-Jun Xu, Qi-Han Zheng, Xu-wen Zheng, Hua Wang.

**Writing – review & editing:** Mao-Bing Chen.
